# Suicide along the Australian coast: Exploring the epidemiology and risk factors

**DOI:** 10.1371/journal.pone.0251938

**Published:** 2021-05-20

**Authors:** Jasmin C. Lawes, Amy E. Peden, Lyndal Bugeja, Luke Strasiotto, Shane Daw, Richard C. Franklin

**Affiliations:** 1 Surf Life Saving Australia, Bondi Beach, Sydney, New South Wales, Australia; 2 Beach Safety Research Group, School of Biological, Earth and Environmental Sciences, UNSW Sydney, Kensington, New South Wales, Australia; 3 College of Public Health, Medical and Veterinary Sciences, James Cook University, Townsville, Queensland, Australia; 4 School of Population Health, Faculty of Medicine, UNSW Sydney, Kensington, New South Wales, Australia; 5 Department of Forensic Medicine, Monash University, Southbank, Victoria, Australia; 6 Monash Nursing and Midwifery, Monash University, Clayton, Victoria, Australia; Charles Sturt University - Port Macquarie Campus, AUSTRALIA

## Abstract

Suicide is an increasing global concern with multiple risk factors, yet location-based understanding is limited. In Australia, surf lifesavers (SLS) and lifeguards patrol the coast, performing rescues and assisting injured people, including people who suicide. This study is a descriptive epidemiological analysis of Australian coastal suicide deaths. The results will be used to inform training and support surf lifesaving personnel and suicide prevention organisations. This is a population-based cross-sectional study of suicide deaths at Australian coastal locations (between 1 January 2005 and 31 December 2019). Data were sourced from the National Coronial Information System and SLS Australia’s Incident Report Database. Analyses explored decedent, incident, and risk factors by sex and method. Across the study period, there were 666 coastal suicide deaths (71.0% male, 43.4% jumping from high places [X80]). Males were more likely to suicide by other means (hanging, self-poisoning, firearm discharge; n = 145, 83.8%), compared to females who were more likely to suicide by drowning ([X71]; n = 77, 37.7%). In one third (n = 225, 38.3%) toxicology was a contributing factor. The risk of coastal suicides was 10.3 times higher during the seven-days prior to their birthday (p<0.001). Evidence of mental ill health was reported in 61.4% (n = 409) of cases and evidence of suicidal behaviour was reported for 37.4% of decedents (n = 249), more prevalent in females. SLS responded in 10.7% (n = 71) of coastal suicides (most jumps from high places; n = 36, 50.7%). Coastal suicides differ to national trends suggesting that location-based differences should be considered during development of preventative and protective measures, especially at a community level. Accessibility, availability, perceived lethality and symbolic qualities are proposed to influence suicide location decisions. These results will guide support and education strategies for surf lifesaving personnel, contributes to established, ongoing suicide surveillance efforts (including hot-spot identification) and add to the limited literature exploring place-based suicide.

## Introduction

Suicide is estimated to have claimed the lives of 817,000 people worldwide in 2016 [1]. Though global suicide rates are decreasing [1], males, people experiencing socio-economic disadvantage [1], those with psychiatric disorders [2] and those who abuse drugs and alcohol [3] continue to be at increased risk of suicide. Given the unprecedented challenges currently facing the global community (e.g. climate change and a global pandemic) and the impacts that social, environmental, and economic factors can have on public health [4–9], there is a concern that suicidal behaviour will remain high [10]. The World Health Organization (WHO) has provided a framework for effective suicide prevention strategies [11], and highlighted the importance of developing timely and accurate data collection systems. The collation of such databases will strengthen our capacity to monitor suicide events to better understand their characteristics, and assist to identify vulnerable individuals or populations and emerging high-risk situation or locations [10,11].

The risk of suicide is influenced by multiple factors, yet our understanding of the association of suicide with natural environmental locations remains limited. While natural outdoor spaces and coastal proximity have been suggested to be protective against suicide mortality and to promote mental health [9], research has investigated urban vs. rural environments, motels, and national parks as location-based risk factors [8,12–14] which shows location impacts suicide risk. In general, the risk of suicide is reported to be higher in rural environments than their urban counterparts [13,14], although there are some contrasting findings that identify an concentration of suicide in coastal communities [15] and in more urbanised areas [16]. This demonstrates that investigations into environmental and urban/rural associations are growing but highlights the lack of detailed location-based research. The location of suicide can influence its outcome, with potential bystanders, availability and accessibility all hypothesised to be important drivers of suicide method choices [17,18]. While there is a growing body of literature, it is evident that little is known about how, and the extent to which, natural environments (in particular coastal environments) are associated with suicide.

After hanging and jumping from high places, drowning is one of the most lethal methods of suicide [19,20]. Studies of suicidal drowning have reported significant mental illness [21], differences among males and females regarding aquatic location of suicide [21] and lower drug and alcohol concentrations when compared to unintentional drowning [21,22]. Suicidal drowning can occur in a range of aquatic locations, including bathtubs [23], swimming pools [24], and both inland and coastal natural bodies of water [25]. Despite this, details regarding the specific body of water are often insufficient for detailed classification [26]. In the Australian context, drowning, alongside hanging, strangulation and suffocation, self-poisoning, and a jumping from height are common method of suicide among both males and females [25,27]. Between 2006 and 2014, the crude mortality rate for intentional drowning was 0.23 per 100,000 population; with males, older people (in particular those aged 75 years and older), non-Indigenous people and urban dwelling people at significantly increased risk [26]. Reduction in the availability of lethal methods [27] in Australia has seen the overall burden attributed to suicide decline, however intentional drowning rates have remained constant over time [26].

Suicide was ranked as the 14th leading cause of death of Australians in 2018 [28], and the leading cause of death for Australians aged between 15–44 years [28,29]. Australia is an island continent with an extensive coastline deeply embedded within Australian culture [30–32]. Coastal environments are popular and dynamic, with natural coastal environments (e.g. beaches, rocks and offshore waters) common locations for unintentional Australian drowning deaths and other fatalities to occur [31–33]. To this end, surf lifesaving services have been established nationally around the coast to prevent drowning and other incidents [33] as well as a growing body of coastal research examining drowning prevention [31,32]. To the best of our knowledge there have been no detailed epidemiological studies that have extended coastal safety research to suicide within Australia or internationally. A greater understanding and awareness of the circumstances and risk factors surrounding these tragic incidents may facilitate more targeted preventative approaches.

In Australia, our surf lifesaving personnel (comprising volunteer surf lifesavers, paid lifeguards and rescue helicopter teams) are tasked with patrolling the coastline and performing rescues and assistance to people injured or at risk in the coastal environment [33]. While these services were initially established to prevent drowning, the service has been extended over time in response to community needs and now regularly encompasses nearby coastal environments as required, including (but not limited to) beaches and surrounding public amenities (e.g. nearby carparks and sand dunes), rocky cliff locations, nearshore environments, and offshore waters. Sadly, part of this service also involves search and rescue including body retrieval of those who die from suicide, by drowning and other methods, resulting in various causes of death. This role, and the associated demands it can have on a primarily volunteer-based workforce (aged 13 years and above) [34], combined with a call for more recent studies exploring suicidal drowning [35], has prompted this study.

Suicide can have fatal and non-fatal outcomes, with a range of associated morbidity. This study aimed to conduct a descriptive epidemiological analysis of suicide deaths along Australia’s coastline with the primary aim to better inform training and support provision to surf life savers [34], while also contributing to the growing body of literature regarding suicide method to strengthen suicide surveillance and inform preventative efforts.

## Methods

### Study design

A population-based cross-sectional study of suicide at coastal locations in Australia was conducted.

### Inclusion and exclusion criteria

Cases were included in the study that met the following eligibility criteria:

- the incident occurred between 1 January 2005–31 December 2019 (inclusive)
- the incident occurred at a coastal location comprising between onshore and 12 nautical miles (nm) offshore where onshore locations included (but were not limited to) beaches and surrounding public amenities (e.g. nearby carparks and sand dunes), rocky cliff locations; nearshore environments, and offshore waters up to 12 nm
- the incident outcome was death and was notified to the Coroner
- the manner of death was determined by the Coroner as intentional self-harm
- the investigation was completed by the Coroner at the time of data extraction (31^st^ August 2020)
- The deceased was 18 years or older at the time of the incident.

Cases were excluded where the suicide followed a murder, thus removing both the deceased and offender. Cases classified with undetermined intent (n = 7) were also excluded from analyses.

### Data sources

The primary data source for this study was the National Coronial Information System (NCIS), an electronic database of deaths notified to Australian Coroners from July 2000 (2001 in Queensland) and deaths notified to New Zealand Coroners from July 2007. The NCIS is an electronic database for which the Department of Justice and Community Safety as the source organisation of the data. The NCIS contains coded and free-text information on the decedent, the incident and cause and manner of death. The NCIS also includes up to four full text reports: police narrative of circumstances of the death; autopsy report; toxicology report; and coroners’ finding. Data from the NCIS was supplemented with Surf Life Saving Australia’s (SLSA) Surf Guard Incident Report Database (IRD). Surf Guard IRD stores information uploaded by surf lifesaving personnel from incident reports following the provision of medical treatment or search and rescue operations.

### Case identification

Deaths were identified using the key word and “query design” in the NCIS. The cause of death field was searched for the key words “drown*” and “immersion” and a query was performed where the variable “Location Level 2” was classified as “beach, shore, bank of a body of water”, “large area of water”, “stream of water”, “marsh, swamp”, “drain, channel”, “lookout, viewpoint”, “base of precipice, cliff”, “wharf, pier, jetty”, “watercraft” or “boat ramp”. The search results included the variable “intent—completion” and “activity” which enabled records returned to identify cases where the Coroner determined the death was a result of intentional self-harm (hereafter referred to as suicide). A review of the Coroners’ finding for each case returned from the search was reviewed against the inclusion and exclusion criteria. Where a Coroners’ finding was not available in the NCIS, the police narrative of circumstances of the death and variables: “cause of death”, “intent–completion”, “location level 2” and “activity” were reviewed against the inclusion criteria.

### Data extraction and management

For each death that met the inclusion criteria, the NCIS record was reviewed and information was extracted on the decedent, the incident and relevant historical and proximate circumstances. Information on the decedent comprised: age; sex; and birth continent. Incident factors comprised: year, season, month of year, day of week and time of day; public holiday; date of personal significance; location; remoteness (coded in accordance with the Australian Statistical Geographical Classification—Remoteness Areas); and external cause of death (coded in accordance with the WHO’s International Classification of Diseases, Tenth Revision [ICD-10]). Relevant historical and proximate circumstances were reviewed to identify risk factors which comprised: evidence of alcohol or drug consumption; evidence of mental ill health; and evidence of a history of suicidal behaviour. Raw data were only accessed, managed and analysed by JL and LS in accordance with ethical agreements.

Where an ICD-10 external cause code was not recorded in the NCIS, two members of the research team (LB, JL) independently assigned a code based on the cause of death. Conflicts in classification of the ICD-10 codes was resolved by a third member of the research team (AP). Where the incident time of day was estimated as within the preceding 24 hours, the midpoint was used and for cases where the incident time of day was estimated as greater than 24 hours, it was recorded as unknown. Evidence of mental ill health (diagnosed or suspected) and evidence of suicidal behaviour (ideation, previous attempts and self-harm) was identified from the reports attached to the NCIS (where available).

To determine the presence and impact of important dates (such as public holidays and birthdays) on suicide, the following coding was undertaken. Firstly, the date of the incident was identified from the NCIS database. If the specific date of the incident was not known, the following steps were undertaken:

- If the date range was greater than three days it was classified as unknown and excluded from the date analyses.
- If the date range was less than three days and there was a specific incident on the day last seen indicating that was most likely the day of death (such as the decedent sending a suicide text message to somebody) then that was used as the incident day.
- If there was no information clarifying when the incident occurred and the time frame was less than three days, the average day was used.

The decedent’s date of birth as listed in NCIS was used. If the date of birth was unknown this case was excluded from the data analyses. A separate database was compiled of all public holidays for each state for each year within the study period, including the holiday date, name and year of the holiday.

### Data analysis

#### Epidemiological statistical analyses

Data was exported into SPSS and univariate and chi square analyses were performed. Decedent, incident and historical and proximate circumstances were analysed by sex and ICD-10 cause of death (X80 jumping from high places, X71 drowning-related suicides, and other). Where multiple variables within the one category were analysed, a modified Bonferroni correction [36] was applied. Due to the number of cases still under investigation for 2019 (54.8%), trend analyses were conducted on cases occurring between years 2005–2018.

#### Important dates analyses

Important dates data were cleaned and analysed in R version 3.6.1 [37]. The package ‘tidyverse’ was used to clean the datasets [38]. For the important dates analyses, a new variable was created in the public holiday dataset, to find the date one week before the public holiday and one week after, creating a date range. This process was repeated in the fatality dataset. The public holiday dataset was then matched to the incident date on the fatality dataset to identify the number of drownings on a public holiday. Similarly, the number of deaths one week before and one week after the public holiday was established by testing if the incident date well within the week range before/after (but not including the date of) the public holiday.

Relative risk ratios were then performed in R using the ‘epitools’ package [39]. The number of deaths that occurred in the 15-year period on a public holiday/seven days before/seven days after and the number of deaths not occurring on those dates was compared to the total number public holidays/days seven days before/seven days after experienced by the Australian public in that time period and the total number of days experienced by the Australian public not on those dates. The decedent’s birthday was matched to the incident date to identify the number of drownings occurring on a birthday. Similarly, the number of deaths one week before and one week after a birthday was established by testing if the incident date within the week range before/after (but not including the date of) the birthday. Relative risk ratios were then performed in R using the ‘epitools’ package [39].

#### Toxicological analyses

The presence of alcohol and drugs was identified using data from toxicology reports (where available) and was examined using a previously established method [32]. The only difference to this previous method was that therapeutic levels of drugs used to treat mental ill health were only included for this if they were used in combination with alcohol. Otherwise, therapeutic levels of drugs used to treat mental ill health were classified as non-contributory even though drugs such as benzodiazepines physiologically cause sedation through their GABA agonistic action, as at a therapeutic level they should decrease the burden of mental health conditions and decrease suicide. It should be noted that a side effect of psychiatric medication in some patients can be a short-term increase in suicidal ideation. However, the overall use of prescription medication to treat mental ill health will normally either decreases suicides [even when first initiating treatment; 40] or have minimal effect [such as some antipsychotics when treating schizophrenia; 41–43]. One potential exception to this may be the use of benzodiazepines, however the cause of this relationship is considered to be partially attributed to withdrawal and overdose, neither of which would have therapeutic levels and would already be covered under this methodology [44].

Drug concentration data was also analysed for those decedents whose death was attributable to toxicology. Due to small sample sizes for individual drugs, drugs were grouped by class and the concentration per class was displayed as a ratio for the serum drug concentration in the decedent compared to the minimum drug concentration that could have impacted death either due to physiological effect on behaviour or toxic levels. If a decedent had multiple drugs of the same class at physiological or toxic levels the higher number was used for analysis. Cases were excluded for concentration analysis where the toxicology, autopsy or coroner’s report clarified that the sample suffered from decomposition.

The overall counts of alcohol and drug concentrations were analysed using relative risk ratios using the R package ‘epitools’ [39] conducted in in R (R V.3.6.1). The drug concentrations were calculated using non-parametric permutational ANOVAs with the package ‘lmperm’ [45], which increases the power of analysis for data with decreased sample size and has no assumptions of underlying distributions, is robust for unequal variances, sample sizes and outliers [45,46].

### Ethics

This study was conducted with ethics approval from the Department of Justice and Community Safety Human Research Ethics Committee (JHREC CF/19/21185).

## Results

**Fig 1:**
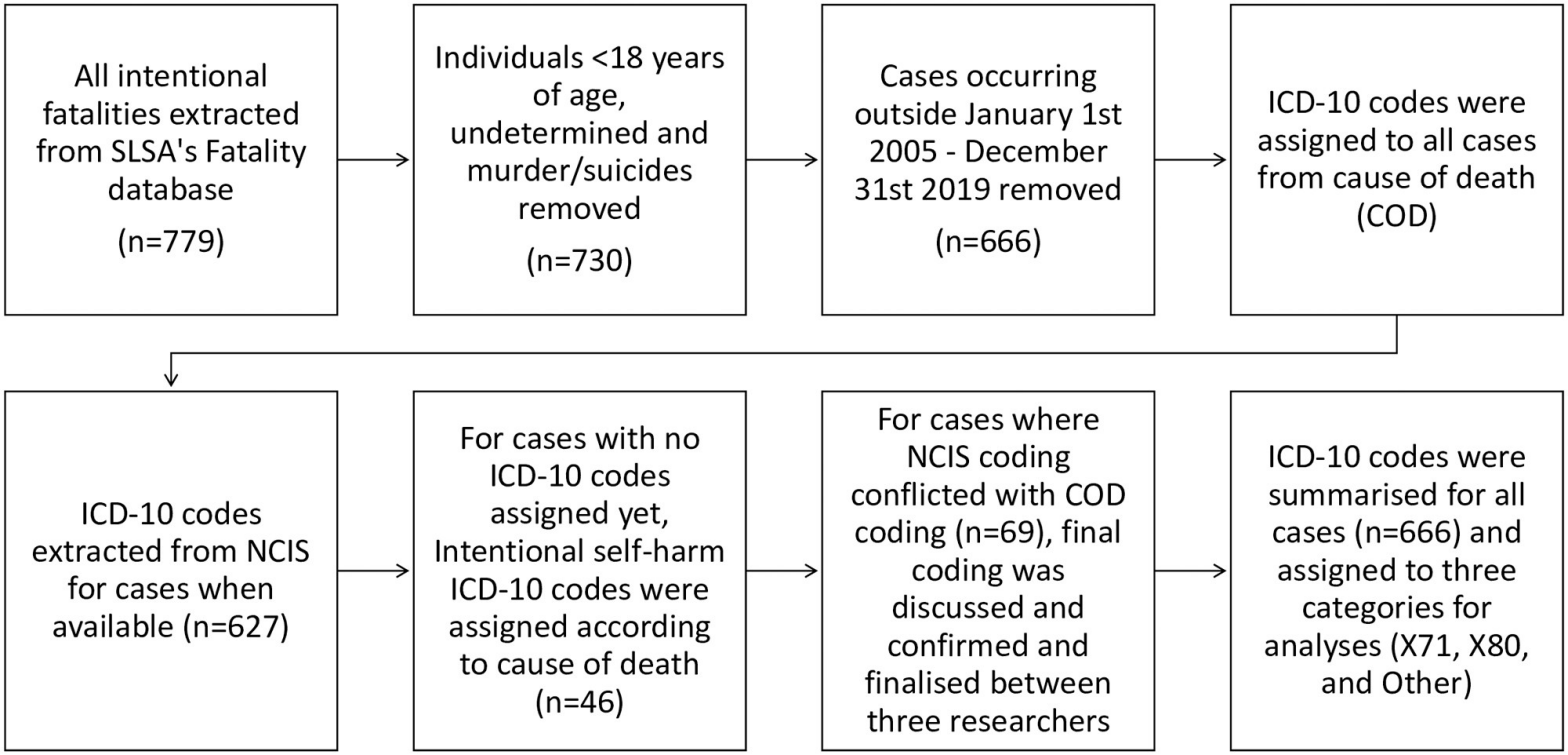
Data inclusion and coding process.

**Fig 2:**
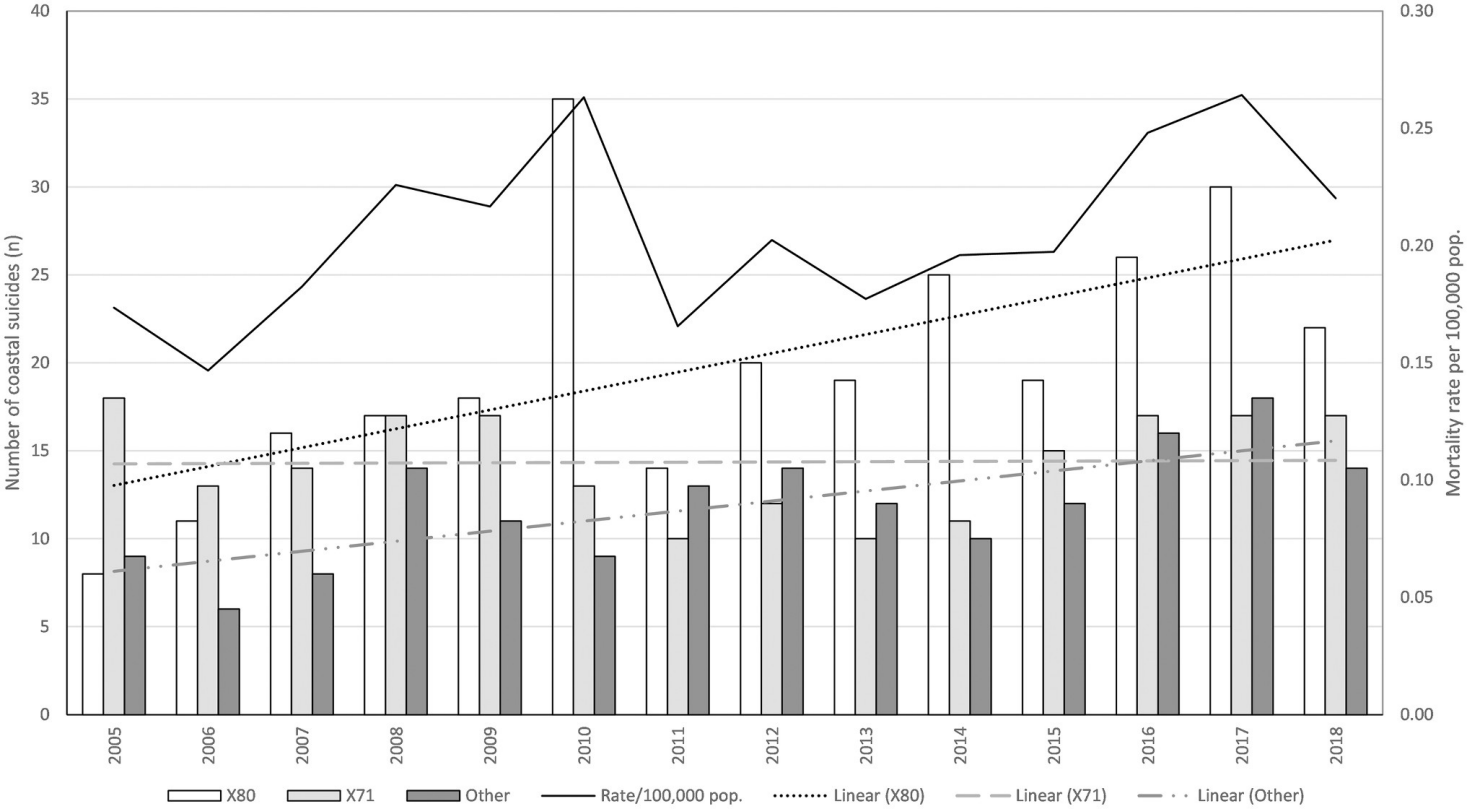
Number and linear trends over time by intentional cause of death (jumping from a height [X80], drowning [X71] and other) at coastal locations, and overall mortality rate for coastal suicides by 100,000 population, Australia 2005–2018.

Between 1 January 2005–31 December 2019 there were 666 suicide deaths in coastal locations (Fig 1). This equates to an annual average of 44 suicide deaths and an average mortality rate of 0.2 per 100,000 population). The leading causes of death were jumping from high places (X80; n = 289, 43.4%), followed by drowning and submersion (X71; n = 204, 30.6%). All other causes were then grouped into a combined category ‘Other’ (n = 173, 26.0%). Within the ‘Other’ category, the main notable causes of death were hanging (n = 61, 9.2%), self-poisoning (n = 38,5.7%) and firearm discharge (n = 18, 2.7%). Annual trends (2005–18) demonstrate that coastal suicide deaths increased over the study period with the number of deaths from jumping from high places (X80) increasing the most (y = 1.0725x + 11.956; R^2^ = 0.3837), followed by other causes (y = 0.5714x + 7.5714; R^2^ = 0.5317) and then drowning-related (X71; y = 0.0154x + 14.424; R^2^ = 0.007; Fig 2).

Coastal suicide deaths predominantly involved males (n = 473, 71.0%) more than females (n = 193, 29.0%) and the largest proportion were classified as ‘jump from a height’ (n = 289, 43.4%), which was significantly more common than the other means (n = 173, 26.0%; Table 1). Jump from a height was the most common method for both males (n = 201, 42.5%) and females (n = 88, 45.6%) and for most age groups (except those aged 70 years or older (n = 24, 30.4%; Table 1). Males were more likely to suicide by other means at coastal locations (n = 145, 83.8%), compared to females who were more likely to drown (n = 77, 37.7%; Table 3). The age groups, 40–54 (n = 195, 29.3%) and 25–39 year olds (n = 195, 29.3%) represented the highest cohorts, with no differences by sex (Table 1). Age groups did differ by method with comparisons revealing 25–39 year olds were more likely to suicide by other means (n = 70, 35.9%) and less likely to drown (n = 36, 18.5%), while the opposite was observed for individuals 70 years or older (drowning n = 45, 57.0%; jump from a high place n = 24, 30.4%; other n = 10, 12.7%; Table 1). The majority were Australian-born (n = 391, 58.7%) and there was no difference observed by birth continent by sex (Table 1). Continent of birth differed by method where Australian-born individuals were more likely to jump from a height (n = 196, 50.1%), Asian-born individuals were more likely to drown (n = 21, 56.8%) and oceanic-born individuals were more likely to suicide by other means (n = 14, 51.9%) and less likely to jump from a height (n = 5, 18.5%; Table 1).

**Table 1:** Person-related variables by sex and ICD-10 code mechanism of cause of death in coastal environments, X^2^ (p-value), Australia, 2005–2019.

Variable	Total	Total		Sex				Test statistic (p-value)	ICD-10 Code						Test statistic (p-value)
				Male		Female			X80		X71		Other		
		N	%	n	%	n	%		n	%	n	%	n	%	
		666	100.0	**473**	**71.0**	**193**	**29.0**	χ^2^ = 117.72 **(p<0.001)**	**289**	**43.4**	204	30.6	173	26.0	χ^2^ = 32.49 **(p<0.001)**
**ICD-10 Code**	X80	289	43.6	201	69.6	88	30.4	χ^2^ = 21.68 **(p < 0.001)**	-	-	-	-	-	-	-
	X71	204	30.6	127	62.3	**77**	**37.7**		-	-	-	-	-	-	-
	Other	173	26.0	145	83.8	28	16.2		-	-	-	-	-	-	-
**Age**	18–24	69	10.4	49	71.0	20	29.0	χ^2^ = 6.089 (p = 0.193)	38	55.1	15	21.7	16	23.2	χ^2^ = 49.418 **(p<0.001)**
	25–39	195	29.3	148	75.9	47	24.1		89	45.6	36	18.5	70	35.9	
	40–54	195	29.3	127	65.1	68	34.9		82	42.1	64	32.8	49	25.1	
	55–69	128	19.2	90	70.3	38	29.7		56	43.	44	34.	28	21.	
	70+	79	11.9	59	74.7	20	25.3		24	30.4	45	57.0	10	12.7	
**Birth Continent**	Australia	391	58.7	275	70.3	116	29.7	χ^2^ = 6.946 (p = 0.321)	196	50.1	87	22.3	108	27.6	χ^2^ = 39.59 **(p<0.001)**
	Europe	70	10.5	54	77.1	16	22.9		28	40.0	28	40.0	14	20.0	
	Asia	37	5.6	22	59.5	15	40.5		9	24.3	21	56.8	7	18.9	
	Oceania	27	4.1	21	77.8	6	22.2		5	18.5	8	29.6	14	51.9	
	Africa	NP	<5.0	9	NP	NP	NP		7	NP	NP	NP	NP	NP	
	North America	NP	<5.0	5	NP	NP	NP		NP	NP	NP	NP	NP	NP	
	Latin America	NP	0.5	NP	NP	NP	NP		NP	NP	NP	NP	NP	NP	
	Unknown*	120	18.0	86	70.9	34	29.1	*	40	33.3	54	45.0	26	21.7	*

Note:

* indicates unknowns were excluded from analyses. After Bonferroni adjustment, significance was deemed at p = 0.004. Bold type indicates significance. NP Not presented due to ethical agreements.

Location did not differ by sex but did differ by method. Jump from a height was significantly more common at rocky cliff locations (n = 270, 81.1%) and less common at other locations, while drowning-related suicide was higher at bays (n = 36, 72.0%) and beaches (n = 90, 47.1%) and lower at rock/cliff locations (n = 32, 9.6%; Table 2). Other means of suicide were higher at beaches (n = 92, 48.2%) and offshore (n = 12, 66.7%), and lower at rock/cliff locations (n = 31, 9.3%; Table 2).

**Table 2:** Environmental characteristics by sex and ICD-10 code mechanism of cause of death in coastal environments X^2^ (p value), Australia, 2005–2019.

Variable		Total (N = 666)		Sex				Test statistic (p-value)	ICD-10 Code						Test statistic (p-value)
				Male (n = 473)		Female (n = 193)			X80 (n = 289)		X71 (n = 204)		Other (n = 173)		
	Categories	N	%	N	%	N	%		N	%	n	%	N	%	
**Location**	Rock/Cliff	333	50.0	225	67.6	108	32.4	χ^2^ = 17.839 (p = 0.013)	270	81.1	32	9.6	31	9.3	χ^2^ = 422.04 **(p<0.001)**
	Beach	191	28.7	136	71.	55	28.8		9	4.7	90	47.1	92	48.2	
	Bay	50	7.5	36	72.0	14	28.0		**NP**	**<15**	36	72.0	10	20.0	
	Jetty	24	3.6	22	91.7	NP	<15		**NP**	**<15**	13	54.2	9	37.5	
	River/Creek	21	3.2	15	71.4	6	28.6		**NP**	**<15**	12	57.1	7	33.3	
	Port/Marina	20	3.0	18	90.0	NP	<15		**NP**	**<15**	11	55.0	9	45.0	
	Offshore	18	2.7	NP	NP	NP	NP		**NP**	**<15**	NP	22.2	12	66.7	
	Other	8	1.2	NP	NP	NP	NP		NP	NP	5	62.5	NP	NP	
	Unknown*	NP	<5.0	NP	0.0	NP	100.0	*	NP	NP	NP	NP	NP	NP	*
**Remoteness**	RA1 –Major Cities	446	67.0	308	69.1	138	30.9	χ^2^ = 6.131 (p = 0.294)	232	52.0	140	31.4	74	16.6	χ^2^ = 116.658 **(p<0.001)**
	RA2 –Inner Regional	113	17.0	81	71.7	32	28.3		42	37.2	39	34.5	32	28.3	
	RA3 –Outer Regional	67	10.1	50	74.6	17	25.4		11	16.4	18	26.9	38	56.7	
	RA4 –Remote	15	2.3	13	86.7	2	13.3		**NP**	**NP**	NP	**NP**	**11**	**73.3**	
	RA5 –Very Remote	14	2.1	12	85.7	2	14.3		NP	NP	NP	NP	**12**	**85.7**	
	Offshore	9	1.4	8	88.9	1	11.1		NP	NP	NP	NP	6	66.7	
	Unknown*	NP	<5.0	NP	NP	NP	NP	*	NP	NP	NP	NP	NP	NP	*

Note:

* indicates unknowns were excluded from analyses. After Bonferroni adjustment, significance was deemed at p = 0.004. Bold type indicates significance. NP Not presented due to ethical agreements.

Remoteness of location did not differ by sex but did differ by method. Jump from a height was significantly more common in major cities (n = 232, 52.0%) and less common in outer regional (n = 11, 16.4%; Table 2) and remote locations (6.3%). Other means were more common at outer regional (n = 38, 56.7%), remote (n = 11, 73.3%) and very remote (n = 12, 85.7%) locations but less likely in major cities (n = 74, 16.6%; Table 2).

While there is some variation in the number of deaths by months, season, day and time of day there were no differences for month by sex or method (S1 Table). Even so, February (n = 74, 11.1%) and January (n = 60, 9.0%) recorded the highest numbers. Jumping from a height was highest in February, October, January and March; suicide by drowning was highest in February, May and July; while other means were highest in September, February and January (S1 Table). One in five occurred on Mondays (n = 122, 18.3%), followed by Saturdays (n = 109, 16.4%) and Thursdays (n = 103, 15.5%). Most occurred over summer months (n = 192, 28.8%) but occurred consistently throughout the year (S1 Table). Time of day was not significant, with coastal suicides occurring consistently throughout the day, however there were a large number of incidents for which time of incident was unknown (n = 159, 23.9%; S1 Table).

Toxicology results were available for 587 (87.2%) coastal suicides, for which 225 (38.3%) reported the presence of toxicants (predominantly alcohol and drugs; Table 3). Broadly, the presence of toxicants did not differ between males and females (38.3%). Alcohol (n = 136, 23.2%) and drugs (n = 151, 25.7%) each contributed to one in four coastal suicides. Males were less likely to die under the influence of both drugs and alcohol (RR = 0.64, p = 0.076) and more likely to after consumption of alcohol alone (RR = 1.73, p = 0.054), however these were not statistically significant. All other risk ratios for alcohol and general drug use were non-significant (Table 3).

**Table 3:** Summary of broad toxicology contributions (alcohol, drugs, combined alcohol and drugs) to coastal suicide deaths.

Toxicology	Male (n)	Female (n)	Total (n)	RR	95% CI	p-value
Alcohol	60	14	74	1.73	1–3.01	0.054
Drugs	62	27	79	0.93	0.61–1.41	0.7
Alcohol and Drugs _a_	38	24	62	0.64	0.4–1.03	0.076
*Alcohol Total*	98	38	136	1.04	0.75–1.45	0.83
Alcohol > 0.05	92	35	127	1.08	0.76–1.52	0.74
*Drugs Total*	100	51	151	0.79	0.6–1.06	0.119
Other	NP	NP	NP	NA		
Unknown	58	28	86	NA		
None	254	104	358	NA		
Total (known) _b_	418	169	587			
Grand Total _b_	476	197	673			

^a^ Included in categorical totals ‘*Alcohol Total’* and ‘*Drugs Total’*.

^b^ Excludes categorical totals ‘*Alcohol Total*’ and ‘*Drug Total’*.

NA Not analysed; NP Not presented due to ethical agreements.

Blood alcohol concentration did not differ by sex (Pseudo-F_1,114_ = 1.245, p = 0.32; S2 Table), while drug concentrations were higher in females (Pseudo-F_1,114_ = 2.091, p = 0.046; S2 Table).

Benzodiazepines/sedatives were the most common drug reported in 24% of deaths, often in combination with drugs of other classes (Table 4). Prescription drugs were less likely to be present in male suicide deaths (RR 0.62, p < 0.01), in particular prescription opiates (RR 0.44, p = 0.041) and anti-depressants (RR = 0.53, p = 0.0377). Prescription drug concentrations were lower in males than females (Pseudo-F_1,87_ = 1.77, p = 0.048; S3 Table). Specifically, antidepressant concentrations were significantly lower for males (Pseudo-F_1,38_ = 1.28, p = 0.03). There were no significant relationships for illicit drugs or other classes of drugs (Table 4; S3 Table).

**Table 4:** Detailed summary of drug contributions to coastal suicide deaths.

Drug Category	Drug Class	Male (n)	Female (n)	Total (n)	RR	95% CI	p-value
**Illicit**	Total	32	10	42	1.3	0.65–2.59	0.6
	Amphetamines	11	NP	NP	0.9	0.29–2.78	1
	Cannabis	15	NP	NP	1.22	0.41–3.63	0.61
**Prescription**	Total	67	44	111	0.62	0.44–0.87	**0.0076**
	Benzodiazepines/sedatives	27	16	43	0.68	0.38–1.24	0.22
	Opiates (Prescription)	13	12	25	0.44	0.2–0.95	**0.041**
	Anti-depressants	25	19	44	0.53	0.3–0.95	**0.0377**
	Anti-psychotics	7	6	13	0.41	0.14–0.12	0.21
**Other**		8	0	8			
**None**		313	118	431			
**Unknown**		35	18	53			
**Total (known)**		441	179	620			
**Grand Total**		476	197	673			

NP Not presented due to ethical agreements.

The risk of coastal suicide deaths occurring on an individual’s birthday did not differ (RR = 1.02; p = 0.62) but did however in the surrounding time period. The risk was 10.3 times higher during the seven-day period prior to their birthday (RR = 10.3; 95% CI = 5.54–19.16; p < 0.001). A similar trend for the week following their birthday was also observed, however this was not statistically significant (RR = 3.09; 95% CI = 1–9.6 p = 0.08). Public holidays (and the seven-day period pre- and post-) did not influence the risk of coastal suicides occurring (S4 Table).

Evidence of mental ill health was reported in almost two-thirds of cases (n = 409, 61.4%) and evidence of suicidal behaviour reported in over one-third of cases (n = 249, 37.4%; Table 5). Evidence of both mental ill health and suicidal behaviour was reported in a third of cases (n = 225, 33.8%), but is potentially higher given the number of unknown cases (n = 334, 50.2%; Table 5).

**Table 5:** Evidence of mental ill health (MH), evidence of suicidal behaviours (SB) and their co-occurrence, by sex and ICD-10 code mechanism of cause of death in coastal environments X^2^ (p value), Australia, 2005–2019.

Variable	Categories	Total (N = 666)		Sex					ICD-10 Code						
				Male (n = 473)		Female (n = 193)		Test statistic (P-value)	X80 (n = 289)		X71 (n = 204)		Other (n = 173)		Test statistic (P-value)
		N	%	N	%	N	%		N	%	N	%	N	%	
**Evidence of Mental ill health**	Yes	409	61.4	275	67.2	134	32.8	χ^2^ = 11.122 **(p = 0.004)**	182	44.5	122	29.8	105	25.7	χ^2^ = 0.798 p = 0.939
	None	66	9.9	57	86.4	9	13.6		28	42.4	22	33.3	16	24.2	
	Unknown	191	28.6	141	73.8	50	26.2		79	41.4	60	31.4	52	27.2	
**Evidence of suicidal behaviours**	Yes	249	37.4	**161**	**64.7**	**88**	**35.3**	χ^2^ = 8.967 **(p = 0.011)**	102	41.0	86	34.5	61	24.5	χ^2^ = 3.068 p = 0.546
	None	96	14.4	76	79.2	20	20.8		45	46.9	26	27.1	25	26.0	
	Unknown	321	48.1	236	73.5	85	26.5		142	44.2	92	28.7	87	27.1	
**Co-occurrence**	MH only	43	6.5	32	74.7	11	25.6	χ^2^ = 14.395 **(p = 0.002)**	23	53.5	8	18.6	12	27.9	χ^2^ = 5.199 (p = 0.519)
	SB only	11	1.7	11	100.0	0	0		5	45.5	3	27.3	3	27.3	
	MH + SB	225	33.8	**140**	**62.2**	**85**	37.8		91	40.4	80	35.6	54	24.0	
	Neither	53	8.0	**45**	**84.9**	**8**	15.1		21	39.6	19	35.8	13	24.5	
	Unknown*	334	50.2	245	73.4	89	26.6	*	149	44.2	95	28.2	93	27.6	*

Note: After Bonferroni adjustment, significance was deemed at p = 0.0125. Bold type indicates significance.

There were no differences observed for evidence of mental ill health or suicidal behaviours and their co-occurrence by method of suicide but there were significant differences observed by sex (Table 5). Incidents involving males were significantly less likely to report evidence of mental ill health or suicidal behaviours, while females were more likely (Table 5). No evidence of mental ill health was more likely to be reported for male suicides than females (Table 5). Evidence of both mental ill health and suicidal behaviour was more likely for females while males were more likely to report neither mental ill health nor suicidal behaviours (Table 5).

Emergency services were reported to respond to most coastal suicides (n = 548, 81.4%) as were bystanders (n = 427, 64.1%; Table 6). Surf lifesaving services were reported to have been involved in the response efforts for 10.7% (n = 71) of coastal suicides, over half of which were jump from a height (n = 36, 50.7%) followed by drowning (n = 18, 25.4%) and other means (n = 17, 23.9%; Table 6).

**Table 6:** Reported responders to coastal suicides, by sex and ICD-10 code mechanism of cause of death in coastal environments X^2^ (p value), Australia, 2005–2019.

Responders	Total (N = 666)		Sex					ICD-10 Code						
			Male (n = 473)		Female (n = 193)		Test statistic (P-value)	X80 (n = 289)		X71 (n = 204)		Other (n = 173)		Test statistic (P-value)
	N	%	N	%	N	%		N	%	n	%	N	%	
Surf lifesaving services	71	10.7	55	77.5	16	22.5	χ^2^ = 1.462 p = 0.481	36	50.7	18	25.4	17	23.9	χ^2^ = 1.773 p = 0.777
Emergency services	543	81.5	384	70.7	157	28.9		233	42.9	167	30.8	143	26.3	
Bystander	427	64.1	308	72.1	119	27.9		183	42.9	131	30.7	113	26.5	
Unknown*	73	11.0	52	71.2	21	28.8	*	29	39.7	25	34.2	19	26.0	*

Note:

* indicates unknowns were excluded from analyses.

## Discussion

This is the first exploration of Australian coastal suicides and the first study to examine the coast specifically as a location with respect to suicide deaths. There were 666 suicide deaths at coastal locations over the study period with rates increasing over time and jumping from high places the most common cause. Coastal areas have been suggested to be areas of heightened risk for suicides [8] with a greater knowledge of local geography needed to better understand and attempt to mitigate this risk. Being an island continent, coastal areas are prominent in Australia with 85% of the population living within 50km of the coast [47]. Australians also have a unique relationship with the coast [48] and there is a need to better understand suicide deaths at these locations to help develop better response as-well as prevention and mitigation strategies.

Australia has one of the most extensive surf lifesaving services globally and surf lifesaving and support operations (i.e. rescue helicopters, jet rescue and offshore rescue boats) were known to be involved in the response efforts of 11% of coastal suicide deaths in Australia, interestingly over half (51%) of which were due to jumps from high places. This is likely to be an underestimation since information was unknown about the circumstance surround the suicide deaths in 11% of cases, and complete details about response effort was rarely available. Surf Life Saving Australia is an iconic organisation that provides prevention and rescue life saving services across Australian beaches and nearby coastal areas, with members and first responders attending a range of coastal incidents, including those which may be intentional self-harm [34]. As an organisation, Surf Life Saving Australia has an obligation to understand these situations better to provide the appropriate level of support to their members [34]. While exposure levels are not explored in this paper, this remains a significant finding since a large proportion of the lifesaving service workforce are volunteers working on the weekend and paid lifeguards during weekdays, and are trained with a focus on drowning-related incidents. The results from this study will guide support and education strategies for surf lifesaving and lifeguard personnel, as well as contribute to established, ongoing suicide surveillance efforts, research and prevention.

### Describing Australian coastal suicides

Suicide is a complex issue, with extensive impacts that extend beyond the numbers to people, families and communities [49–52]. This study is the first to describe coastal suicide deaths in Australia framed by suicide method and sex of decedents. In general, the number of coastal suicide deaths in Australia are increasing and the number of suicide deaths was approximately three times higher in males than females, consistent with national suicide reporting [50,51]. Most decedents were Australian-born (59%, n = 391), however 23% were born overseas, and for 18% birth continent was unknown. This is higher than the recently reported proportion of Australian residents that were born overseas [30% in 2019; 53], supporting the framing of coastal environments as ‘lethal locations’ that are purposefully travelled to by decedents for increased anonymity and reduced chances of being interrupted [17,18]. While the highest number of coastal suicides occurred over the summer months and on Mondays, coastal suicides occurred consistently throughout the year, on any day and at any time during the day.

The circumstances under which people choose to take their own life are complex and often there is no single determining factor why an individual dies by suicide [54]. Locations away from home and certain method choices are suggested to have higher rates of lethality due to lower risk of intervention [17,18]. Jumping from a height was the most common method of suicide used in almost half of all coastal suicide deaths (43%), followed by drowning and then other means. The proportional use of these methods deviates considerably from the national trends [55] in which the most common suicide methods (hanging and self-poisoning) were less common in coastal suicides, whereas nationally jumping from a high place is fourth-highest and drowning and submersion is ninth [55]. Jumping from heights and suicidal drowning were more common in coastal environments [56], since the coastline may combine accessibility with availability due to the increased presence of the ocean or naturally elevated structures such as rocky cliffs [56]. In this study, rocky cliff locations recorded the highest number of coastal suicide deaths (50%) followed by beaches and bays, which would directly correlate with jumping from height and drowning methods. These results identify coastal environments as lethal locations but suggest a greater measure of lethality at rocky cliffs, beaches and bays, e.g. jumping from heights at a cliff may be considered more lethal than self-poisoning at home which could be more easily interrupted or have a delayed outcome or require more pre-planning. These results indicate that coastal suicides and their intervention or prevention strategies may require a different, or more localised, suite of responses and strategies with respect to suicides that occur inland or at home.

The costs of suicide to individuals, communities and society are extensive, with certain groups identified as being at greater risk than others [49,50]. Suicide is the leading cause of death for 15–44 year olds, especially men [10,50], with research investigating and supporting preventative strategies that are both community-based and broader, national public health approaches [50,57,58]. While this study excluded those under 18 years of age, our results support this with decedents aged between 25–59 representing the largest cohort, comprising almost two-thirds of all coastal suicides (59%). This trend has also been reported in the United States where suicide rates among adults aged 24–44 years old have exceeded those reported for older adults [65 years or older; 59]. This stage of adult life often encompasses intense employment or relationship development in combination with new responsibilities (i.e. parenthood) and is therefore likely to have multiple co-occurring stressors. For example, unemployment has been strongly associated with male suicide rates in Australia [especially those between 25–34 and 55–64 years of age; 29], however the relationship differed depending on the age group and the duration of unemployment–highlighting the challenge of deciphering causal factors within suicide deaths. The complexity surrounding suicide prevention, and the interrelated contributing factors within these incidents, suggests that individual and community-level strategies that aim to reduce suicidal behaviours through the incorporation of multiple protective factors are required to make communities and individuals more resilient and to reduce suicidal behaviour [29].

### Fatal coastal suicide methods

Locations away from home are considered more lethal [18,60], but lethality also differs with the choice of suicide method. Practical aspects, particularly availability and accessibility, are hypothesised to be important drivers of suicide method choice [61,62] and essentially underpin prevention strategies that restrict access of hotspot areas [e.g. bridges; 63]. The impact of psychological and cultural factors on method choice have also been recognised [56] with highly-publicised suicide events, often at formidable locations, touted to increase the number of subsequent suicides that intentionally re-enact the publicised suicide in the months following the initial fatality [60,64]. The motives behind these incidents are suggested to be more symbolic or personally driven rather than by accessibility or availability alone.

Coastal areas in Australia comprise impressive rocky cliffs against wild unpredictable oceans, and given the coast is considered to be part of the Australian psyche, it is plausible that coastal environments combine practical drivers (accessibility and availability) with more symbolic motivators behind suicidal choices that are made by the decedent. The familiarity of, and the perceived capacity to effectively carry out the method, may also influence method choice [64]. For example, familiarity with firing a firearm would be generally lower across Australia since there is strict legislation in place regarding firearm ownership. This legislation significantly limits firearm availability and familiarity, and reduces the rate of Australian firearm-related suicides, in contrast with those recorded in firearm heavy countries [e.g. the United States; 65,66]. Since research that specifically investigates suicide at the individual method level is limited [but see 21,26,35,67–70], this section discusses coastal suicide deaths in the context of suicide method.

### Jumping from a high place (X80)

Jumping from a high place was the most common coastal suicide method recorded and was increasing more than other methods. It was the highest cause of death for males, females, most age groups (except for 25–39 year olds and those aged 70 years or older) and was more prevalent in Australian-born decedents. Suicide by jumping from a height was significantly higher at rocky cliff locations and major cities which is likely to reflect the availability of naturally elevated locations along coastal environments with a higher perception of lethality and access by large numbers of people [71,72]. Elevated locations are also prevalent in outer-regional and remote coastal locations, yet jumping from high places was lower in comparison to other means. Since rates of suicide are generally considered to be higher in rural settings [73,74], this suggests that other drivers (e.g. population size, social determinants, or greater accessibility to other means such as firearms) may influence suicide method choice more in regional and remote areas [73]. This highlights a difference in the prevalence of coastal suicides by jumping in relation to regionality, and that suicide prevention strategies to circumvent suicide by jumping maybe be more relevant for coastal locations in major cities than in regional or remote areas.

Suicide by jumping from high places can involve extremely traumatic mechanisms of injury resulting in significant trauma and is strongly associated with an increased incidence of death, which some may consider more lethal than other means [71]. Jumping may also be considered as a more public act of suicide [75,76] and, by some, as a method that reflects a greater intent to die [75]. However, it should be acknowledged that the reasons behind chosen suicide methods are complex and apparently less-lethal means of suicide do not necessarily reflect a lesser desire to die, nor that there is consideration about lethality. With respect to jumping, some naturally elevated sites have gained a level of notoriety as suicide ‘hotspots’. Practical characteristics including accessibility, perceived lethality and symbolic qualities such as media attention or natural features (e.g. overlooking water) are suggested to contribute to this notoriety [72]. Much research has demonstrated that restricting access to these notorious sites leads to a reduction in suicide deaths from jumps at those sites [75,77], with little evidence to suggest substitution of location to alternative sites [63,77]. Since it is impractical and undesirable to restrict access to Australia’s extensive coastline, the implementation of exclusion strategies that target identified coastal suicide hotspots could provide an opportunity for the individual in distress to reconsider their actions [77]. While a national strategy is important to guide prevention efforts, these results emphasise the need for localised strategies that integrate local knowledge and build awareness of protective factors at the community level.

### Drowning and submersion (X71)

Drowning-related suicides increased over the study period but at a lower rate than other methods. Decedents aged 70 years and older were more likely to suicide by drowning (57% of all fatalities of people aged 70 years and over), consistent with previous findings [67], while decedents aged 25–39 years were less likely (19%). This may be related to increasing intentional drowning among elderly males and a preference for less violent means among elderly females [67].

Decedents born in Asia were more likely to intentionally drown (57% of all Asian-born coastal suicide deaths), while Australian-born decedents were less likely (22% of all fatalities). This may be linked to cultural practices around suicide, with drowning considered to be a preferred method among people from Asia, in particular females [78]. This preference for suicide by drowning could also be linked to poorer swimming skills and higher drowning rates commonly seen in Asian nations [79,80], since a fatal outcome would be more likely if the decedent does not know how to swim.

Linked to the location-based focus of this study, is research that indicates that drowning and jumping are methods of suicide more commonly seen in natural environments [56]. In this study of coastal environments in Australia, drowning was the second leading method of intentional death (31%), after jumping from a high place (43%). Of note, drowning-related suicides were more likely to occur in bays (72% of all bay suicides) and at beaches (47% of all beach suicides). This provides further strength to the need to consider suicide risk (and associated prevention strategies) from a location perspective [8,22,56], including building awareness of suicidal drowning and the development of suicide-response training by surf lifesaving volunteers.

### Other methods in coastal suicides

The ‘other’ means used in coastal suicide deaths included several different methods with hanging, self-poisoning and firearm discharge being the top three causes of death (combined totalling 17% of all coastal suicides). The number of suicide deaths that incorporate other means increased over time, but given the dominance of jumping and drowning as suicide methods at coastal locations, these other means are comparatively low, especially when compared to national trends [55]. Other means of suicide were higher at beaches and offshore waters, locations which would provide greater opportunity for a range of methods that take into account the geography or nature of the environment [64]. For example, it could be considered easier to find a secluded beach with trees and sand dunes to suicide privately in a seemingly less violent manner by poisoning or hanging. Suicide by other means was higher for oceanic-born decedents and more common in outer regional, remote and very remote areas, suggesting that social and economic determinants may be greater drivers of suicide method choice in these communities. Differences in access may also explain this result, especially given that firearm use in Australia is strongly linked to rural locations [81] and regional and remote communities would be more familiar with discharging a firearm [64], therefore increasing its availability as a suicide method compared to urban communities. These results highlight the diversity within coastal suicides and emphasise that prevention strategies may be more effective if developed and implemented at a community level.

### Risk factors of coastal suicides

Coastal suicides were higher in males than females, consistent with nationally reported trends [50,51], otherwise differences observed by sex in this study related to evidence of mental health and suicide behaviour reported across many of the incidents. Evidence of mental ill health or a history of suicidal behaviours was reported less for incidents involving males, while a greater proportion of female suicide deaths reported a higher incidence of these behaviours, both individually and in combination. While it is well established that males are less likely to seek help, interpretation of our results must be considered with caution as they do not establish this since it may not have been investigated or reported in the coronial documents. This result is however consistent with national reports where suicidal behaviour is more prevalent in females, while males suicide deaths are more numerous [51]. Evidence presented in the National Survey of Mental Health and Wellbeing (2007) suggests that mental ill health and suicidal behaviours (e.g. ideations, self-harm and previous attempts) are widespread in the community, with an estimated impact on 2.1 million Australians [82]. This suggests that behavioural results from this study are probably more reflective of national suicide patterns and unlikely to be location or method specific.

Birthdays can be emotional times which could have associated triggers, which would be worth acknowledging their potential influence. The risk of coastal suicide was more than 10 times higher during the seven-day period prior to their birthday with a similar trend for the week following their birthday. This result contradicts previous findings where no birthday effects were found [83,84], but supports research from Japan where birthdays, especially those celebrating significant milestones, can increase suicide risk [85,86]. These findings further acknowledge the influence of cultural and social factors in suicide surveillance. While this result is unlikely to be linked to location or method choices within suicide, it is important with respect to implementing more protective measures to high-risk individuals in the period leading up to their birthday. A strategy that implements this would be more effective at the individual level, but could potentially be used at higher levels (i.e. community or national) to prompt professional services to check in around high-risk times (i.e. with medical professionals or within databases where personal information is retained after previous records of suicidal behaviours).

Alcohol and drugs are known suicide risk factors, due to their propensity to impair decision making, dramatically influence our response capacity and impact on our emotional state [87]. Moreover, it has been proposed that suicides in natural areas are more likely to involve softer drugs (alcohol and prescription drugs) instead of illicit drugs [56], a hypothesis that is largely supported by our results. Nearly 40% of coastal suicides with known toxicology results were under the influence of alcohol and/or drugs, in spite of the conservative toxicological inclusion strategy implemented in this study where therapeutic doses of psychiatric medications were excluded. This result is comparable to other suicide research [88], but higher than toxicology findings reported in unintentional coastal drowning deaths (24%) and other unintentional coastal fatalities [22%; 33]. Men were more likely to have consumed alcohol, with an average blood alcohol concentration of 0.18 g/100mL (S2 Table), more than three times the legal driving limit. A similar average blood alcohol concentration was found for females (0.22 g/100mL), but there were fewer cases. Alcohol is a known depressant so understanding its role in suicides is significant, these results indicate that alcohol use in coastal suicides is similar to national trends and is not location specific.

Prescriptions drugs played a greater role in female suicide deaths at coastal locations, largely due to increased contribution of anti-depressants and prescription opioids. This result supports previous research where females had a higher prevalence of both anti-depressant and opioid use in suicide [89,90]. In this study, females had a higher serum concentration of antidepressants than males, which could be due to differences in metabolic enzymes [91]. This result has also been shown in naturalistic clinical settings [91] and, given the higher uptake of these prescription drugs due to increased rates of diagnosis [92], it has been proposed that females may exhibit higher tolerance to anti-depressants [92] and therefore may use higher doses. Females are also reported to have an increased incidence of chronic pain compared to males and may manage this pain with prescription opioids [93]. This, alongside higher prevalence of mental ill health conditions such as depression and anxiety [94] and an increased likelihood to receive treatment by women [and prescribed medication; 95,96], could explain the increased prescription drug concentrations found in female coastal suicide deaths.

### Implications

Understanding suicide by location will help develop location and community specific prevention strategies and ensure local knowledge is incorporated into these strategies. Coastal locations are unique, especially in Australia, where there is a workforce (volunteer and paid) who patrol many of the beaches and nearby coastal areas. This workforce is called on to rescue people in trouble, help in the search and rescue of a missing person and also retrieve bodies [33,34]. In the course of their work, they also respond to incidents occurring in the coastal environment but outside of the water (i.e. on rock platforms or in beach carparks). Surf lifesavers, lifeguards and other first responders are often required to attend to intentional incidents, in many cases without knowing the incident was intentional. The adverse impacts of such job-related occupational stressors among first responders, such as post-traumatic stress disorder (PTSD), suicidal thoughts and behaviours, are well-documented [93–100], but as yet unknown for Surf Life Saving [34]. Surf Life Saving Australia, as an organisation, has an obligation to better understand these situations and ensure its members are provided with necessary and appropriate training and equipment to attend such incidents [34]. It is also important to ensure the workforce is adequately supported during and after such incidents, using evidence-based postvention support strategies. Research to better understand impacts of trauma on surf lifesavers while on patrol is currently underway by Surf Life Saving Australia [34].

As part of a broader commitment to saving lives, this present study has identified an opportunity for Surf Life Saving to collaborate with suicide prevention organisations through various means (such as research and information sharing) to identify locations with a high incidence of suicide to guide prevention interventions [77]. The diversity within coastal suicides highlight the importance of integrating local knowledge and community values within national suicide prevention strategies. While it is important to have an overarching national strategy to guide prevention, building awareness and understanding at the community or local level can create greater ownership and therefore implementation of protective measures that could be community or location specific. With the identification of notorious suicide hotspots and a greater understanding of suicide risk factors, the community (including the surf life saving workforce) will be better equipped to not only respond to such incidents, but also to recognise and prevent them from occurring as well as helping to develop localised prevention strategies.

### Strengths and limitations

This paper is novel and makes the first steps towards better understanding suicides along the Australian coast. The use of multiple population-based data sources, including the NCIS and Surf Life Saving’s IRD provides rich detail enabling the identification of risk factors to guide prevention efforts of this complex cause of death. There are, however, limitations associated with this study. Data fields on cases still under coronial investigation are subject to change. At the time of analysis, 97.3% (n = 648) of cases of interest were closed. Cases in more recent years were less likely to have intent completion coding, as such, 2019 data were removed from temporal trend analysis. Some variables are poorly reported within the coronial system, such as birth continent and time of incident, leading to large numbers of unknowns. Similarly, incomplete information regarding the rescue or body retrieval effort and the decedent’s history of mental health and suicidal behaviours also represents limitations of this study and areas of improvement for coronial services. Although out of scope for this study was an examination of the residential or tourist status of overseas-born decedents, the initial findings reported in this study support the need for future investigation into the propensity for distances travelled to a particular suicide location.

There were several cases that did not have intentional self-harm ICD-10 codes as their underlying cause of death despite the precipitating factors being an act of suicide. This highlights a challenge with respect to coding deaths and a concern of omitting data when using ICD-10 codes alone for analyses. Similarly, due to the systematic nature of coronial data collection related to cause of death, protective factors are not included in the data. It is important to note that the role they play in an individual’s life is extremely pertinent [52]. Further work is required to increase awareness across data custodians of the importance of complete records and their use post classification.

With respect to the dates of significance analyses, all public holidays were used, as opposed to placing a value judgement on a particular day that may be more significant (i.e. Christmas Day). For incidents which occurred within a date range, the public holidays or birthdays may have been missed, as they may have occurred in the week before or after. This study has not undertaken an exhaustive analysis of psychosocial factors, and it must be acknowledged that suicidal triggers differ at the individual level, therefore the findings of this study represent generalisations that should be interpreted with caution.

The toxicological analyses do not account for complex interactions between drugs or in combination with alcohol (for example when multiple central nervous system depressants such as opioids, benzodiazepines and alcohol are combined an individual is at an increased risk of overdose due to the additive interactions between the substances [101]. Similarly, the concentration analysis in this study assumes a linear dose response and does not account for an individual’s tolerance to a drug or any potential drug redistribution after death.

Toxicological analyses conducted in this paper are conservative with therapeutic levels classified as non-contributary as therapeutic doses of psychiatric mediation generally decreases the chance of suicide [40]. The authors would like to acknowledge that therapeutic levels of drugs used to treat mental health conditions can cause a range of side effects in patients, which can increase suicide risk. However, it would since mental health medication are generally effective at decreasing mental health symptoms and preventing suicide, it would be erroneous to classify the therapeutic presence of any as being contributory [40].

## Conclusion

Suicide is a leading cause of death in Australia. This is the first epidemiological exploration into suicide deaths at coastal locations and considerably adds to the limited body of research exploring place-based suicide. Understanding suicide by location will help to develop location-specific prevention strategies and ensure that local knowledge is incorporated into the implementation of these strategies. The results of this study have highlighted the importance of community-level understanding since the challenges differ between communities. For example, jumping from high places is more prominent in major cities at rocky/cliff locations, while other means for suicide would be better targeted in regional or remote communities. This research has also confirmed that access, availability (including familiarity), cultural and social determinants can all drive suicide method selection but this may differ with location.

The involvement of historical and proximate risk factors were reflective of national suicide trends as opposed to location or method specific, emphasising the need to reduce stigma surrounding mental health and suicide to build national awareness and acceptance, with the period leading to birthdays potentially important times to increase protective measures around high-risk individuals. This research has provided novel insights into a previously unexplored aspect of the significant Surf Life Saving workforce (which is largely comprised of volunteers) and the exposure of personnel to these incidents through involvement in response efforts. In particular, our results highlighted that many intentional incidents that surf life saving services respond to are as a result of jumping from a high place and not drowning. This will allow for Surf Life Saving Australia as an organisation to ensure its personnel are adequately provisioned and trained for suicide response, and are supported during and after incidents, including access to relevant and helpful support networks.

## Supporting information

S1 Table Extra environmental variables by sex and ICD-10 code mechanism of cause of death in coastal environments Χ2 (p value), Australia, 2005–2019. (XLSX)

S2 Table Detailed summary of drug contributions to Australian coastal suicides. (XLSX)

S3 Table Broad summary of toxicological contributions (alcohol, drugs, combined alcohol and drugs) to coastal intentional fatalities. (XLSX)

S4 Table The relative risk of Important Days (Public holidays, birthdays) and the seven-day period pre- or post-important day (inclusive) on coastal suicides. Bold type indicates significance.
(XLSX)
